# Predicting Impacts of Climate Change on the Aboveground Carbon Sequestration Rate of a Temperate Forest in Northeastern China

**DOI:** 10.1371/journal.pone.0096157

**Published:** 2014-04-24

**Authors:** Jun Ma, Yuanman Hu, Rencang Bu, Yu Chang, Huawei Deng, Qin Qin

**Affiliations:** 1 State Key Laboratory of Forest and Soil Ecology, Institute of Applied Ecology, Chinese Academy of Sciences, Shenyang, People’s Republic of China; 2 University of Chinese Academy of Sciences, Beijing, People’s Republic of China; 3 Shengli Oilfield's Shengli Engineering co., LTD, Dongying, People’s Republic of China; The Ohio State University, United States of America

## Abstract

The aboveground carbon sequestration rate (ACSR) reflects the influence of climate change on forest dynamics. To reveal the long-term effects of climate change on forest succession and carbon sequestration, a forest landscape succession and disturbance model (LANDIS Pro7.0) was used to simulate the ACSR of a temperate forest at the community and species levels in northeastern China based on both current and predicted climatic data. On the community level, the ACSR of mixed Korean pine hardwood forests and mixed larch hardwood forests, fluctuated during the entire simulation, while a large decline of ACSR emerged in interim of simulation in spruce-fir forest and aspen-white birch forests, respectively. On the species level, the ACSR of all conifers declined greatly around 2070s except for Korean pine. The ACSR of dominant hardwoods in the Lesser Khingan Mountains area, such as Manchurian ash, Amur cork, black elm, and ribbed birch fluctuated with broad ranges, respectively. Pioneer species experienced a sharp decline around 2080s, and they would finally disappear in the simulation. The differences of the ACSR among various climates were mainly identified in mixed Korean pine hardwood forests, in all conifers, and in a few hardwoods in the last quarter of simulation. These results indicate that climate warming can influence the ACSR in the Lesser Khingan Mountains area, and the largest impact commonly emerged in the A2 scenario. The ACSR of coniferous species experienced higher impact by climate change than that of deciduous species.

## Introduction

Forests store the most carbon of any unit of the terrestrial ecosystem [1], [2], and the majority of the carbon sequestrated is held in woody biomass [3]. Forests play a vital role in climate change mitigation [4] and water conservation [5], [6]. Also, forests can avoid soil erosion [7], although the impact of vegetation cover on soil erosion is not straight forward [8], [9]. They provide many ecological services including biodiversity protection, a supply of wood and fiber, and functions related to tourism and recreation [10]. The potential capacity of forests to sequester carbon will obviously influence the future balance of global carbon flux; however, this potential is largely determined by the rate of carbon sequestration occurring in forests [11]. The aboveground carbon sequestration rate (ACSR) of forests is an important index reflecting the usefulness of forest ecosystems to humans. The future dynamics of ACSR has aroused many concerns, especially when one considers the impacts of climate change.

Human activity has altered the concentration of atmospheric carbon dioxide in a way that will create serious consequences such as warmer climates and irregular patterns of precipitation [12]–[14]. Emissions of greenhouse gas that continue at or above current levels are likely to cause additional climatic warming in the future, and this will transform some processes related to forest carbon sequestration such as the productivity, species distribution, and large alterations in nature disturbance regimes [15]. Also, the extension of the growing season and increased rates of photosynthesis which are caused by climate change will enhance forest growth rates [16]. Climate change can change tree species migration patterns [17], which can further affect forest carbon sequestration [18]. The net primary productivity (NPP) of tree species as well as their competitiveness can be changed by the alteration of climatic conditions such as temperature, precipitation, and solar irradiation [19], [20], and the forest ACSR will experience parallel impacts of higher temperatures [21]. In addition, the changing climate will lead to changes in disturbance regimes that will diminish the process of carbon sequestration to a large extent. This makes exploring the rate and potential capacity of forest carbon sequestration necessary [22], [23].

Many studies focus on the process of forest carbon sequestration under climate change, and most of which have researched the climate change impact on forest composition and carbon accumulation [24]–[27] and the responses of forest growth to altered climates [21], [28], [29]. However, relatively little research has explored the speed of forest carbon sequestration, which is important to those developing forest management policies.

**Figure 1:**
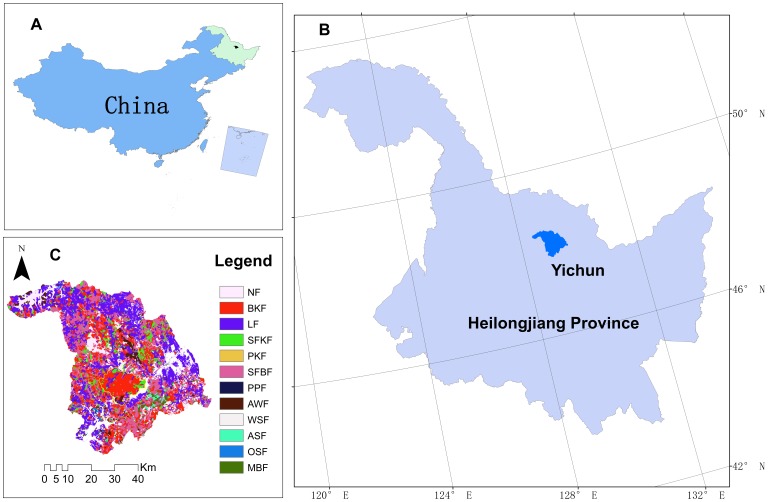
Location of the study area and the distribution of the forest communities. A: Heilongjiang Province in Northeast China; B: study area in Heilongjiang Province; C: The distribution of forest communities in Lesser Khingan Mountains. NF: no forest zone, BKF: Broad-leaved Korean pine forest, LF: Larch conifer forest, SFKF: Spruce-fir Korean pine forest, PKF: Pinus sylvestris-Korean pine conifer forest, SFBF: Spruce-fir broad-leaved forest, PPF: Planted pinus sylvestris forest, AWF: Aspen-white birch forest, WSF: White birch softwood forest, ASF: Aspen softwood forest, OSF: Oak softwood forest, MBF: Mixed broad-leaved forest.

Globally, temperate forest is a widely distributed forest type, and its carbon flux has been significantly altered by the changing climate [30]–[32]. The Lesser Khingan Mountains lie in a transitional region between a cold temperate and a moderate zone and these mountains are covered by typical temperate forests. A variety of vegetation and forest communities can be found in this area (**Fig. 1**), including coniferous forests, mixed broad-leaved conifer forests, and deciduous broad-leaved forests. In the past decade, many research studies related to the impact of climate change on forest ecosystems have been conducted in this area [33]–[35]. However, the dynamics of forest ACSR under climate change scenarios is still unclear. Rational forest management policies, which are designed to maintain sustainable productivity in the future, need to carefully explore the dynamics of forest ACSR. By assessing and understanding the future status of forest carbon sequestration under different climate change scenarios, foresters can make well-designed policies related to forest management.

Quantifying the complex effects on forests caused by global climate and land use changes has proved difficult [26], but we can use ecological models to simulate the forests dynamics. Many previous studies have proved that ecological models have an ability to estimate historical as well as future forest carbon pool dynamics [36], [37]. Forest landscape succession and disturbance (LANDIS) model is a spatially explicit forest landscape model capable of simulating forest succession under multiple natural and anthropogenic disturbance regimes based on the current species distribution, age cohorts, and individuals [38], [39]. Many studies [17], [26], [39]–[41] about forest dynamics under different conditions in North America and China have proved that it has the ability to detect the effects of alternative future climate scenarios on forests [26].

In this study, we couple projected meteorological data using Earth System Models, LANDIS Pro7.0 model and logistics model to simulate the response of forest ACSR to climate change. The objectives of this study were to (1) explore the dynamics of the ACSR in four main communities and fourteen tree species of a temperate forest in the Lesser Khingan Mountains for 200 years starting from 2000, (2) analyze statistical discrepancies found in the different effects of various climate change scenarios on forest ACSR, and (3) provide useful suggestions on how to carry out forest management in a changing climate.

## Methods

### Study Area

Our study area (**Fig. 1**) is located in the northern part of the Lesser Khingan Mountain region near the city of Yi Chun. This area extends across 47.85°–48.05°N, 128.43°–129.62°E, is covered by three forestry bureaus, includes a nature reserve zone, and has total area of about 315,000 ha. Dark brown soil, homogeneously distributed in this area, constitutes the typical soil of this region. The elevation ranges between 400 m and 600 m. The temperate continental monsoon climate experiences cold, long winters (mean January temperature, –25°C) while summers are warm and transitory (mean July temperature, 21°C). The average annual precipitation (550–700 mm) mostly falls from June to August.

The Lesser Khingan Mountains lie in a transitional zone between a cold and a moderate temperate zone. Several conifers and soft-hard woods coexist in this typical temperate forest. Common species include Korean pine (*Pinus koraiensis*), spruce (*Picea koraiensis* and *P. jezoensis*), Khingan fir (*Abies nephrolepis*), larch (*Larix gmelinii*), Manchurian ash (*Fraxinus mandshurica*), Amur cork (*Phellodendron amurense*), Mongolia oak (*Quercus mongolica*), black elm (*Ulmus japonica*), mono maple (*Acer mono*), ribbed birch (*Betula costata*), black birch (*Betula davurica*), Amur linden (*Tilia amurensis*), white birch (*Betula platyphylla*), aspen (*Populus davidiana*). Korean pine is the regionally dominant species, while spruce and Khingan fir are dominant only in high elevation areas.

Four representative communities occur in the study area: mixed Korean pine hardwood forests, spruce-fir forests, mixed larch hardwood forests, and aspen-white birch forests. These four communities are common vegetation types in the Lesser Khingan Mountain area. The entire region has suffered severe deforestation except in the Fenglin Natural Reserve.

### LANDIS Model

LANDIS is a dynamic forest landscape model simulating forest succession, seed dispersal, species establishment, and various types of disturbance such as wind, fire, and timber harvesting [39], [42], [43]. LANDIS Pro 7.0 is derived from an earlier version of LANDIS, in which the landscape is represented as a grid of cells. The cell size can be set from 10 m × 10 m to as large as 500 m × 500 m. This model can simulate changes over long temporal (e.g. >100 years) and at large spatial scales (e.g. >107 ha). Several species were contained in every cell, and LANDIS Pro 7.0 model grouped trees into different species age cohorts. Besides, the model keeps track of the number of trees for each species age cohorts in all cells [44]. The modeled landscape was divided into several land types according to altitude, slope, climatic conditions and other environmental factors. Species establishment coefficients (SEC) (ranging from 0 to 1.0), which are an important input parameter in a LANDIS model, quantify whether a specific land type favors or works against the establishment of a selected species. Similar SECs would be developed in modeling of the same land type [43].

LANDIS Pro 7.0 simulates seedling establishment, growth, death, regeneration, random mortality, and vegetative reproduction on the basis of SEC and species vital attributes (**Table 1**) at the scale of a single modeled cell [45]. A detailed description of these species’ physiological process can be found and consulted in the LANDIS Pro 7.0 user’s guide [44]. An important advance of the latest LANDIS version is that the number of tree for each species age cohort was added in the model. This makes it easy for us to calculate the aboveground biomass of each tree as well as the total biomass of the forest. At a landscape scale, LANDIS simulates spatial processes such as seed dispersal and seedling establishment [17]. Seed dispersal simulates the seed travel process based on a species’ effective and maximum seed dispersal distance. A seedling establishment algorithm starts to work when seeds reached a particular site to decide whether a particular seed can become established based on consideration of other species that occur on the site and the shade tolerance rank of the seeding species relative to the species occupying the site [17], [45]. If a site is occupied by species with a higher shade tolerance (e.g. Korean pine), species with lower shade tolerance (e.g. aspen, white birch) cannot spread into this site. A uniform random number from 0 to 1 will be set to compare with the SEC to decide if seeds can become established. Only when a species’ SEC is greater than the random number can the species become successfully established. That means species with a high SEC will obtain high probabilities of establishment [43].

**Table 1:** Species vital attributes in the Lesser Khingan mountains area, Northeastern China.

Species	LONG	MTR	ST	FT	ESD	MSD	VP	MVP	MD	CCC
**Korean pine (** ***P. koraiensis*** **)**	320	80	4	3	200	600	0	0	130	0.457
**Spruce (** ***P. koraiensis and jezoensis*** **)**	300	30	4	3	80	200	0	0	100	0.447
**Khingan fir (** ***A. nephrolepis*** **)**	300	30	4	3	80	200	0	0	60	0.440
**Larch (** ***L. gmelinii*** **)**	300	20	3	4	80	200	0	0	100	0.454
**Manchuria ash (** ***Fraxinusmandshurica*** **)**	250	40	3	5	400	1000	0.9	50	110	0.433
**Amur cork (** ***P. amurense*** **)**	250	15	3	4	60	300	0.8	60	90	0.443
**Mongolia oak (** ***Q. mongolica*** **)**	320	20	3	5	50	200	1	50	100	0.429
**Black elm (** ***Ulmus japonica*** **)**	250	10	3	3	200	1000	0.5	60	100	0.465
**Mono maple (** ***A. mono*** **)**	200	10	3	3	500	1000	0.5	50	60	0.420
**Ribbed birch (** ***B. costata*** **)**	250	15	3	3	500	4000	0.9	40	90	0.448
**Black birch (** ***B. davurica*** **)**	150	15	3	5	500	4000	0.9	30	50	0.433
**Amur linden (** ***T. amurensis*** **)**	300	15	3	2	80	250	0.8	30	80	0.448
**White birch (** ***B. platyphylla*** **)**	150	15	1	2	500	4000	0.8	50	60	0.451
**Aspen (** ***P. davidiana*** **)**	150	10	1	1	600	5000	0.9	10	60	0.433

LONG: longevity (years); MTR: age of maturity (years); ST: shade tolerance (1–5); FT: fire tolerance (1–5); ESD: effective seeding distance (m); MSD: maximum seeding distance (m); VP: vegetative production probability (0–1); MVP: minimum age of vegetative reproduction (years); MD: maximum diameter at breast height (cm); CCC: carbon content coefficient (0–1).

The species’ growth curve is another essential input parameter used to calculate species biomass in the LANDIS model. In any outputting year, the model reads the corresponding diameter at breast height (DBH) and then outputs biomass by applying allometric growth equations.

### Model Parameterizations

Species’ vital attributes are driving factors of succession and dispersal in LANDIS [45]. Some other input data such as disturbance and management parameters, species composition maps, land type maps, and the species establishment coefficients for each land type are also included in LANDIS [17], [45], [46]. Species’ growth curves and the average number of tree individuals are specifically used to calculate the biomass of each species in the biomass module. Growth of fourteen of the main tree species (four conifer and ten broad-leaf species) in our study area are simulated in LANDIS. The values of these input data were mainly compiled from previous LANDIS parameterization, plot investigation, and consultations with local experts [17], [41], [45], [47], [48]. With the consent of Fenglin Nature Reserve administration, we also investigated some plant plots (Table S1) in the reserve to test whether the parameters of the LANDIS Pro7.0 model were generally reasonable; our field experiments did no harm to the animals and tree species.

We generated an initial species composition map including species as well as age information from a forest stand inventory map and database produced in 2000 (provided by the Forestry Planning and Design Bureau of Heilongjiang Province, 2003). Useful information such as stand boundaries, the relative abundance of canopy species, and the average age of dominant canopy species were obtained from the database. The grid format stand maps with a resolution of 90 m × 90 m were converted from vector format with the goal of reducing computational loads during model simulations. Many previous studies in this area revealed that single species stands occur only during early successional stages [49]–[51]. On most occasions, multiple species occur in a 90 m × 90 m sized pixel. The LANDIS Pro 7.0 model traces species age cohorts in all pixels during the process of succession. The simulated processes of dispersal, establishment, growth, and death were all recorded. So, the model can output species’ aboveground biomass at the end of every simulation time step (we set the time step at 10 years) by calculating biomass using tree biomass equations that are embedded in the LANDIS model.

Logistics models were used to project the SEC for each species, and this model’s input parameters are mainly environmental variables, including slope, aspect, elevation, annual average temperature and precipitation, topographical position index and compound topographical index [17], [52]. We used logistics models to simulate the probability of each species’ occurrence in all cells in the land type every 10 years with the current and future climatic data and output the resulting map. The mean value of the probability of a species appearing in the cells of a specific land type is the final SEC of this species in that land type. We modeled the SECs of 14 species under different climatic conditions from 2000 to 2100 with 10-year increments; the initial SECs in 2000 were generated under current climatic conditions. The SECs after 2100 were assumed to remain stable.

The development of forest and the dynamics of carbon flux were affected by some disturbances such as land use change, CO_2_ fertilization, and outbreak of insect [53], [54]. However, they were not included in full research area, and they are not the main disturbance factors in this region. Therefore, in our study, we only considered climate change and ignored other disturbance or management factors, and we assumed that no fire or other events occurred in this simulation. Three types of climate change scenarios (B1, A1B, and A2) as well as current climatic condition were taken into account, and we attempted to compare different impacts of climatic conditions on the forest ACSR for the next 200 years (2000–2200).

### Climate Data

The current meteorological data were collected from the Northeastern Institute of Weather in China and were compiled for 1961–2005 from 78 weather stations. Regression models were built between spatial position and temperature as well as precipitation. We calculated mean annual temperature and precipitation based on the daily temperature and precipitation. Climate projections generated by the third version of the Canadian Global Coupled Model (CGCM3) were used in this study. Three different scenarios simulating different levels of carbon emissions (B1, A1B, and A2) were adopted to produce future climatic data. The B1 emission scenario represents the lower emission scenario while A1B and A2 scenarios represent the median and higher emission scenario, respectively.

We interpolated the mean annual temperature and precipitation from 78 weather stations distributed throughout Northeastern China into grids with 90 m × 90 m resolution, indicating the distribution of current temperature and precipitation. According to CGCM3, the mean annual temperatures and precipitations of all climate scenarios would increase in first 100 years (2000–2100), and some studies believed the climates would enter into a stable state after 2100 and fluctuate around the level in 2100 [17], [55]. We calculated the annual temperature differences between the future warming and current climate in 2000, as projected by CGCM3, using equation (1):
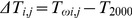
(1)where 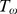 represents temperature under warming climate, 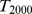 represents temperature in 2000 projected by CGCM3, *i* represents the year (2000< *i* <2100), and j is the decade (2010< *j* <2100 with a 10-year increment); therefore, 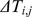 is the climate change for year *i* and decade *j*. The projected mean annual temperature and precipitation of each decade from 2000 to 2100 were obtained when 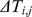 was added into the initial grids. **Table 2** shows the increment of mean annual precipitation and temperature in 2100. The current mean precipitation and temperature were 555.1±24.1 mm, and –0.54±0.48°C, respectively. The final results of climatic data were adopted by the logistics models to output SECs.

**Table 2:** The variation values of average annual precipitation and temperature in 2100 modeled by CGCM3.

Climate scenarios	ΔP (mm)	ΔT (°C)
**B1**	65.03±16.75	1.00±0.07
**A1B**	60.22±7.32	2.00±0.02
**A2**	240.38±17.30	3.21±0.41

ΔP:Increment of precipitation in 2100 modeled by CGCM3; ΔT: Increment of temperature in 2100 modeled by CGCM3.

### Modeling and Analyzing Approaches

This study coupled the LANDIS model and a logistics model to simulate the forest ACSR in the Lesser Khingan Mountains region. The logistics model was used to simulate the species’ physiological response to current and changing climate conditions, and it outputted SECs for each time step corresponding with those of the LANDIS model. Furthermore, the LANDIS model was used to simulate species establishment, succession, and the process of forest carbon sequestration using the SECs obtained from the logistics model under the different climate scenarios. LANDIS Pro 7.0 can read SECs for every simulated time step, so the results output for species’ biomass could reflect the effect of climate change on forests. The current climate and three warming climate scenarios were adopted in this study, and these results were compared.

Carbon content coefficient (CCC) as a vital factor was applied to convert biomass to carbon content. Results from an existing study [56] using CCCs of several main tree species in China’s northeastern forest region were adopted in this study (**Table 1**). We used the variation of carbon content every ten years to represent the forest ACSR; the ACSR of fourteen species as well as four forest communities were analyzed. Equation (2) displayed the formula used to calculate the species’ carbon sequestration rate:
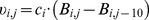
(2)where *i* represents tree species and *j* is the decade (2010< *j* <2200 with a 10-year increment). 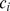 represents the CCC of species *i*. 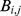 is biomass of species *i* in decade *j*, and 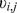 is the average carbon sequestration rate of species *i* in decade *j*. For example, 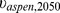 is the average forest carbon sequestration rate of aspen in the period from 2040 to 2050. Five replicas were simulated for each climate scenario to assess model stochasticity, and we took the average values as the final carbon sequestration rate. We divided the total simulation into four time periods (2000–2050, 2060–2100, 2110–2150, and 2160–2200) and used one-way ANOVA to test the hypothesis that differences of ACSR existed among various climate scenarios. The LSD multiple comparison method was used to detect the difference of ACSR under various climate conditions. Furthermore, we conducted T-tests among the 157 measured biomass data from the plots in a natural reserve and the value of each time step’s total biomass output from 2100 to 2200 in our simulation. All statistics were conducted using open resource statistical graphics and computing environment R with *P*<0.05 used as a test of significance [57].

## Results

### ACSR at Community Level

**Figure 2:**
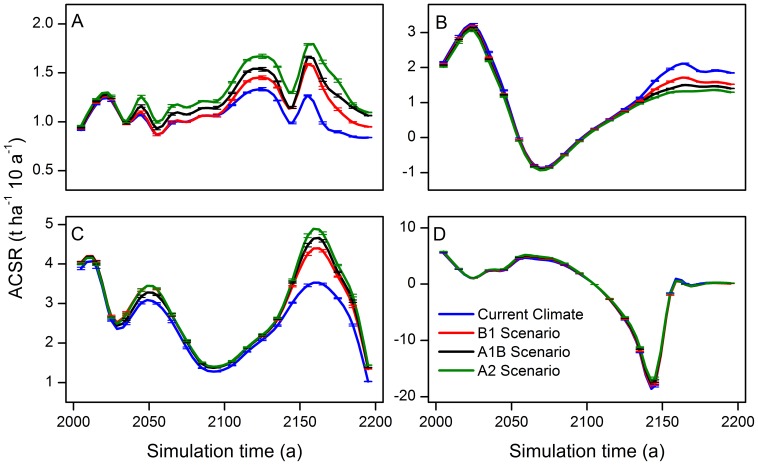
Forest aboveground carbon sequestration rates of different forest communities. A: Mixed Korean pine hardwood forests, B: Spruce-fir forests, C: Mixed larch hardwood forests, and D: Aspen-white birch forests.

Parallel dynamics of the ACSR under current climate conditions and three climate warming scenarios during the simulation were detected in all four forest communities. The ACSR of mixed Korean pine hardwood forests and mixed larch hardwood forests fluctuated during the entire simulation (**Fig. 2A, 2B**). The range of variation of ACSR in the two communities were 0.84–1.87 t ha^–1^ 10 a^–1^ and 1.03–4.78 t ha^–1^ 10 a^–1^, respectively. The ACSR of the two communities under all warming scenarios was always higher than that under current climate.

The ACSR in the spruce-fir forest and aspen-white birch forest communities initially fell, followed by a rising trend (**Fig. 2C, 2D**). However, different beginning time of falling and falling ranges existed in the ACSR of the two communities. The ACSR of spruce-fir forests experienced a short rise before 2030. The minimum values of the ACSR of these two communities were –0.88 t ha^–1^ 10 a^–1^ in the 2070s and –18.02 t ha^–1^ 10 a^–1^ in the 2140s, respectively. In the spruce-fir community, the ACSR under the warming scenarios generally was lower than that under the current climate, especially after the year of 2140. The ACSR of the aspen-white birch community almost tended to zero at the end of the simulation.

### ACSR at Species Level

**Figure 3:**
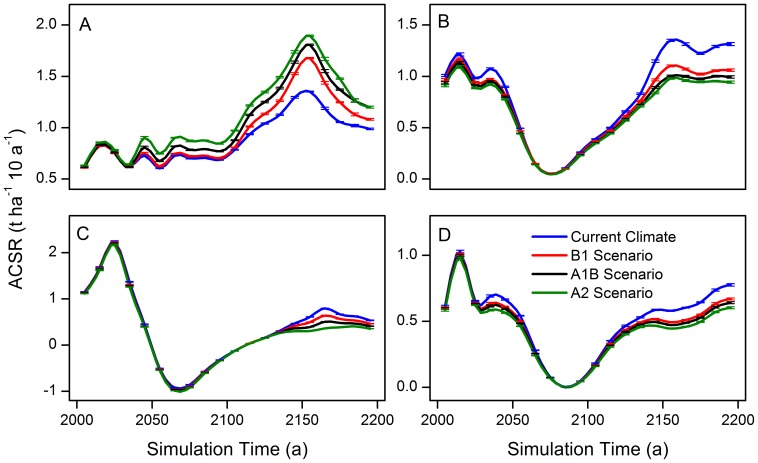
Forest aboveground carbon sequestration rates of four conifers. A: Korean pine, B: Spruce, C: Khingan fir, and D: Larch.

In conifers, the ACSR of the species adapted to a warm-climate, e.g. Korean pine (**Fig. 3A**), fluctuated in the first half of the simulation under current and warming climates and varied in the range of 0.61–0.86 t ha^–1^ 10 a^–1^. The ACSR of spruce, Khingan fir, and larch (**Fig. 3**) had similar dynamics, which displayed a rising trend after an initial falling trend and finally fluctuated after 2140, and they varied in the range of 0.71–1.35 t ha^–1^ 10 a^–1^, 0.26–0.79 t ha^–1^ 10 a^–1^, and 0.44–0.78 t ha^–1^ 10 a^–1^, respectively. Most of them reach a minimum ACSR in the period from 2070 to 2090. The ACSR of the three conifers under warming scenarios were relative lower than that under current climatic conditions.

**Figure 4:**
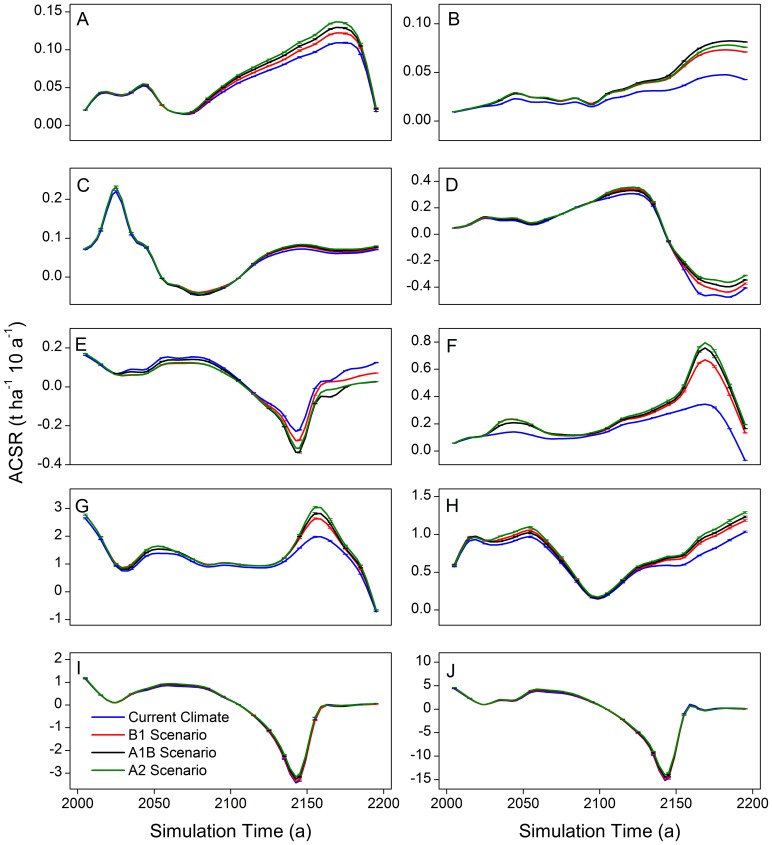
Forest aboveground carbon sequestration rates of ten broad leaved tree species. A: Manchurian ash, B: Amur cork, C: Mongolia oak, D: Mono maple, E: Black birch, F: Black elm, G: Ribbed birch, H: Amur linden, I: Aspen, and J: White birch.

The ACSR of broad-leaved tree species (**Fig. 4**) demonstrated complex dynamics during the simulation. The ACSR of the Manchurian ash, Amur cork tree, black elm, and ribbed birch initially fluctuated and then rose rapidly and finally fell. The ranges of the variations in these four species were 0.02–0.05 t ha^–1^ 10 a^–1^, 0.01–0.03 t ha^–1^ 10 a^–1^, 0.06–0.23 t ha^–1^ 10 a^–1^, and 0.81–1.63 t ha^–1^ 10 a^–1^, respectively. Black elm was the only specie that the ACSR under warming climate lower than that under the current climate. The dynamics of ACSR of Mongolia oak and Amur linden showed a trend of rising before a sharp decline and then recovering later in the simulation. The ACSR of these two species reached a minimum in the 2080s and 2090s, respectively. Two unique patterns of ACSR fluctuations existed for mono maple and black birch in the initial and final periods of the simulation. Initially, the ACSR of these two broad-leaved trees varied in the ranges of 0.05–0.13 t ha^–1^ 10 a^–1^ and 0.06–0.15 t ha^–1^ 10 a^–1^, while the ranges of variation in the last period were –0.47– –0.31 t ha^–1^ 10 a^–1^ and –0.05–0.12 t ha^–1^ 10 a^–1^, respectively. The dynamics of the ACSR of these pioneer species, aspen and white birch, tended to rise after an initial fall and fluctuated around zero after 2160, and the ranges of variation of them before 2080 were 0.10–0.92 t ha^–1^ 10 a^–1^ and 0.96–4.08 t ha^–1^ 10 a^–1^, respectively. The ACSR of these pioneer species reached a minimum in the 2140s simultaneously, and the curve of the ACSR of the two species under all climates nearly overlapped.

### Differences of ACSR among Climate Scenarios

**Figure 5:**
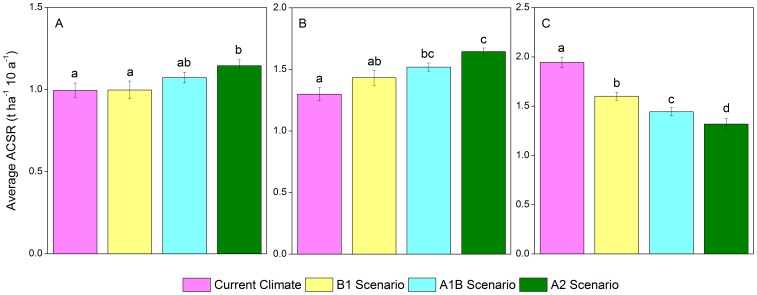
Results of multiple comparisons of the influences on communities’ aboveground carbon sequestration rate. A: Mixed Korean pine hardwood forests in 2060–2100, B: Mixed Korean pine hardwood forests in 2110–2150, and C: Spruce-fir forests in 2160–2200.

According to the results of variance analysis (**Table 3**), significant differences (*P<0.05*) of ACSR existed among the various climate scenarios in mixed Korean pine hardwood forests at 2060–2100 and 2110–2150, and in spruce-fir forests at 2160–2200. No significant difference of the ACSR among those climates was identified in the other two communities. In those two periods, the ACSR of the mixed Korean pine hardwood forests under scenario A2 was significantly higher than that under other climates (**Fig. 5A, 5B**). In the spruce-fir forest community, the ACSR under all warming scenarios were significantly higher than that under current climate scenario, and significant differences existed between any two warming scenarios (**Fig. 5C**).

**Table 3:** ANOVA results of differences among various climate scenarios effect on forest aboveground carbon sequestration rate.

Community with species	2010–2050		2060–2100		2110–2150		2160–2200	
	F	P	F	P	F	P	F	P
**Mixed Korean pine hardwood forests**	0.250	0.860	3.675	**0.035**	6.098	**0.006**	2.813	0.073
Korean pine (*P. koraiensis*)	0.317	0.813	9.618	**0.001**	1.359	0.291	3.210	**0.046**
Manchurian ash (*Fraxinusmandshurica*)	0.019	0.996	0.071	0.975	0.839	0.492	0.180	0.908
Amur corktree (*P. amurense*)	0.163	0.920	3.627	**0.013**	2.451	0.101	20.382	**<0.001**
Mongolia oak (*Q. mongolica*)	0.012	0.998	0.097	0.961	0.045	0.987	4.623	**0.016**
Black elm (*Ulmus japonica*)	0.388	0.763	2.002	0.154	0.753	0.536	2.419	0. 104
Mono maple (*A. mono*)	0.079	0.970	0.005	0.999	0.046	0.986	1.841	0.180
**Spruce-fir forests**	0.058	0.981	0.033	0.992	0.143	0.933	83.712	**<0.001**
Spruce (*P. koraiensis and jezoensis*)	0.919	0.454	0.021	0.996	0.266	0.849	140.216	**<0.001**
Khingan fir (*A. nephrolepis*)	0.009	0.999	0.030	0.993	0.047	0.986	16.843	**<0.001**
**Mixed larch hardwood forests**	0.081	0.969	0.067	0.977	0.098	0.960	0.506	0.684
Larch (*L. gmelinii*)	0.120	0.947	0.015	0.997	0.196	0.898	3.713	**0.034**
Ribbed birch (*B. costata*)	0.035	0.991	0.255	0.856	0.181	0.908	0.137	0.936
Black birch (*B. davurica*)	0.044	0.987	5.366	**0.016**	0.115	0.950	3.354	**0.045**
Amur linden (*T. amurensis*)	0.150	0.928	0.040	0.989	0.104	0.957	1.369	0.288
**Aspen-white birch forests**	0.010	0.999	0.094	0.962	0.007	0.999	0.032	0.992
White birch (*B. platyphylla*)	0.011	0.998	0.093	0.963	0.007	0.999	0.053	0.983
Aspen (*P. davidiana*)	0.008	0.999	0.098	0.960	0.009	0.999	0.015	0.997

df = 4; Bold P values mean the effect of treatment is significant (α = 0.05).

**Figure 6:**
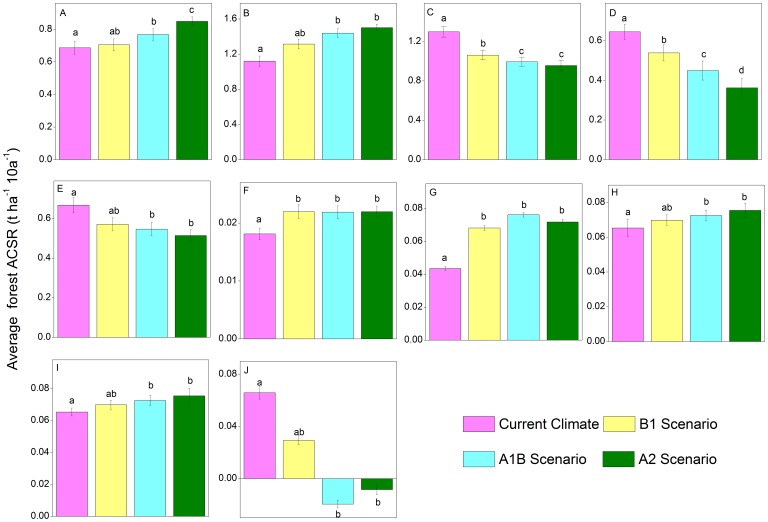
Results of multiple comparisons of the influences on species aboveground carbon sequestration rate. A: Korean pine in 2060–2100, B: Korean pine in 2160–2200, C: Spruce in 21260–2200, D: Khingan fir in 2160–2200, E: Larch in 2160–2200, F: Amur cork in 2060–2100, G: Amur cork in 2160–2200, H: Mongolia oak in 2160–2200, I: Black birch in 2060–2100, and J: Black birch in 2160–2200.

In the species level, only in the periods of 2060–2100 and 2160–2200, significant differences of ACSR existed among various climate scenarios (**Table 3**). The ACSR of all the conifers among all climate scenarios had significant differences (*P<0.05*) in the last quarter (2160–2200) of the simulation, while these differences could be only detected in three hardwoods (**Fig. 6F–J**) (Amur cork tree, Mongolia oak and black elm) in the same period. From 2060 to 2100, the ACSR of Korean pine and Amur cork under the warming scenarios were significantly different from that under the current climate scenario. From 2160 to 2200, the ACSR of the species, with significant differences among climates, under the A1B and A2 scenarios were significantly differ from that under the current climate scenario (**Fig. 6**). Unique differences of ACSR between scenario A1B and A2 was detected in spruce (**Fig. 6C**), while no difference of ACSR was identified between scenario B1 and A1B in all species except spruce and Khingan fir (**Fig. 6D**). Differences of ACSR between the current climate and the scenario B1 were found in spruce, Khingan fir, and Amur cork.

### Spatial Distribution of Forest Biomass

**Figure 7:**
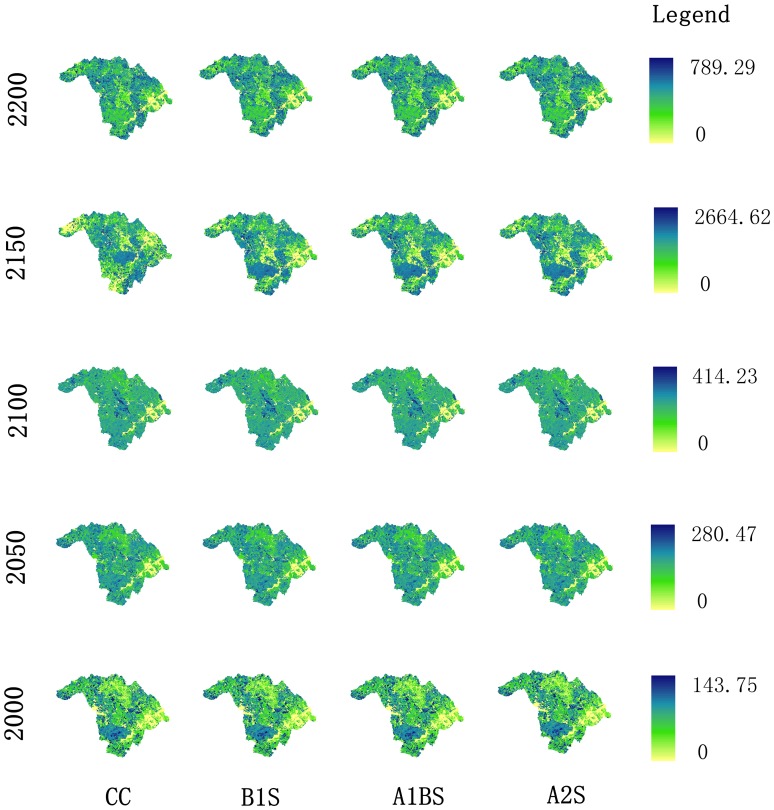
The spatial distribution of forest total biomass under different climates. CC: Current climate, B1S: B1 scenario, A1B: A1B scenario, A2: A2 scenario. Unit: t ha^−1^.

The forest total biomass under current climate and three warming scenarios presented similar spatial patterns in 2000, 2050, 2100, and 2200 (**Fig. 7**). The total forest biomass of this area is generally low except in the Fenglin Natural Reserve. Total biomass constantly accumulated during the simulation, and it becomes hard to detect differences in biomass between the forest reserve zone and other locations. However, large changes in total forest biomass accumulation can be identified during the second half of the simulation. A decrease of simulated total biomass was observed in most areas while it remained at a high level in *Fenglin* Forest Reserve. At the end of the simulation, the total biomass accumulation of the reserve zone was no longer higher than in other zones. The geographic center of the spatial distribution of total biomass tended to move north during the simulation.

## Discussions

The LANDIS Pro 7.0 model is a spatially explicit model, and plays an important role in simulating the processes of germination, seed dispersal and establishment, and growth for various species under various disturbance regimes. The output of species’ biomass makes it possible to explore the dynamics of the forest ACSR.

The trends in the variation of ACSR of all communities and species mostly occurred simultaneously among all the climate scenarios, though some relatively large differences appeared.

At the community level, the effect of multiple climate change scenarios appeared to involve complex processes affecting changes in forest ACSR. The possible explanation of this phenomenon is that usually a small increase in temperature has little impact on the growth of Korean pine, larch, and some other broadleaf species that are dominant species in these communities. This also corresponds with some previous studies [58], [59], which have reported that the forest growth rate will not change until a temperature threshold appears. The temperatures under scenario A2 are likely to exceed this threshold while temperatures under other scenarios are not, and this can lead to ACSR under scenario A2 usually significantly higher than that under others. Moreover, the mixed Korean pine hardwood community is the climax community in this area, and it appears to be well-adapted to the warming climates of the past decades. The general rising trend of ACSR may follow that adaption. This possible inference is in agreement with the local reality of forest distribution.

Significant differences existed between any two climate scenarios in the last quarters of the simulation in the spruce-fir community, and this community was the only one where the ACSR decreased when climate changed from current scenario to warming scenarios. Most likely, this community is vulnerable if the climate becomes warmer, and much greater impact on the ACSR may be caused by higher temperatures. The warming is not good for the growth of Khingan fir. Nonetheless, one study found the biomass of fir increase notably during a 6-year warming trend [60]. However, the increase of biomass is not equal to the increase of ACSR; the biomass will continue to increase as long as the ACSR is greater than 0. Similar patterns in mixed larch hardwood forests in the later period of the simulation were also remarkable; however, the difference was that warming scenarios brought about higher ACSR values. This probably occurred because climate warming created a sharp increase in the abundance of broadleaf species in this community. Broadleaf trees can adapt to higher temperatures better than conifers and this may lead to a northward shift of broadleaf forest [58]. This corresponds well with the northward movement in the spatial distribution of the forest total biomass.

The final decrease of ACSR at the end of the simulation may suggest that the mixed larch hardwood community is a transitional community and will eventually be replaced by hardwood communities. Aspen and white birch are both pioneer species in the aspen-white birch community and they occupy an extensive range in this study area. The sharp decrease of the ACSR in the middle of the simulation may be caused by natural mortality of these species. According to the species vital attributes (**Table 1**), aspen and white birch both live about 150 years; most individuals died around 2150 in the simulations. The fluctuation ACSR around zero in the last decades of the simulation suggests that this community will eventually disappear.

At the species level, the dynamics of the ACSR display a more complex process. The ACSR of most conifers (except Korean pine) in the simulations demonstrated V-shaped alterations, and differences of the ACSR among various climate scenarios emerge late in the simulation. This may denote that the growth process of some conifers, including spruce, Khingan fir, and larch, may essentially stop during the simulation. It has already been proven that many conifers, including spruce and fir, become more vulnerable when climates become warmer [61]. The ACSR of Khingan fir once even dropped below zero, indicating that Khingan fir is not adapted to warm temperature, and the growth would be restrained while climate continues to warm. Global climate has changed as a result of hundreds of years of industrial activity, and some other disturbances such as fire and over exploitation have caused great destruction of forests [62]. This may be the reason why the responses of forests, which have already experienced climate change, appear insensitive to various climate change scenarios. As for Korean pine, increasing temperatures tend to accelerate the increase in ACSR, and its position as a dominant species will undoubtedly be enhanced under warming climatic conditions.

The ACSR of most hardwood species have experienced a rising trend, although some of them (Manchurian ash, black elm, and ribbed birch) decreased at the end of the simulation. This suggests that broadleaf trees have better adaption to warm conditions and grow at a relatively high speed. The final decrease in the ACSR of some broadleaf species likely occurred because they approached their natural longevity at the later time of simulation and began to decline. The smaller initial distribution and lower competitive ability to capture nutrients can well explain why Mono maple has a declining trend of its ACSR [63]. The decline of the ACSR of aspen and white birch is in agreement with the successional nature of aspen-white birch community.

We adopted three climate change scenarios (B1, A1B, and A2) as well as modeled the current climate conditions to detect the different influences of these scenarios on the ACSR. However, significant differences in the ACSR among various scenarios can be only discovered in a small number of communities and species, most of which existed in the last quarter (2160–2200) of the simulation. These facts demonstrate that the effects of various climate change scenarios on the ACSR have no effect in a relatively short time (150 years). This does not mean that no action should be taken to control the current high levels of greenhouse gas emissions, because different climate change scenarios may manifest their effects on the ACSR later than in only 150 years. In other words, a hysteresis phenomenon exists in the process of climate change effects on the forest ACSR in this temperate forest. A slow response time of forests to climate change may be the real reason of this phenomenon.

**Figure 8:**
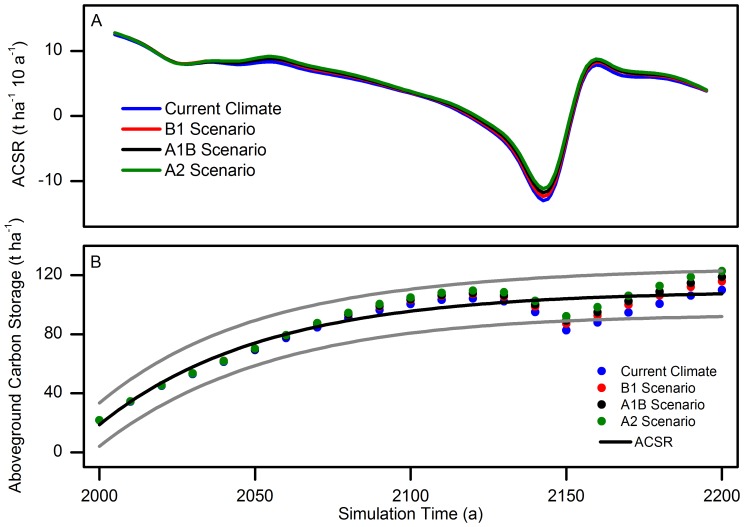
Total forest carbon sequestrations and its rate under various climate scenarios in simulation time. A: Forest total ACSR of different future climates. B: Forest aboveground carbon storage of different future climates. The black line means the average aboveground carbon storage of all possible future climates, and the grey lines indicate the forecasting interval of aboveground carbon storage with the confidence level of 95%.

The stability of a forest ecosystem is expressed in many aspects, such as species composition and biomass accumulation [64]. The carbon sequestration rate and capacity are vital factors reflecting a forest’s ability to sequester carbon. In this study, the distribution of total biomass (**Fig. 7**) on a landscape scale reflects that the carbon sequestration of forest fluctuates during the modeled time period, and the forest displayed an extremely unstable state especially in the last quarter of the simulation. This corresponds with dynamics of the total forest ACSR (**Fig. 8A**). However, the total capacity of the forest for aboveground carbon sequestration maintained at a relative stable level in this landscape (**Fig. 8B**). This indicates that a forest actually reaches a dynamic equilibrium based on the stable state of a forest ecosystem when its biomass accumulation reaching a dynamic equilibrium. The species composition and the spatial distribution of biomass can reflect the status of a forest ecosystem at a large time scale; however, difficulties persist in interpreting the instantaneous status of forest growth. Reflection of the dynamics of forest carbon flux, especially the complex temperate forest, needs indicators that can represent the development process of forest succession. However, traditional indexes such as stand structure, species composition, and biomass are all static indicators. They only reflect the state of forest carbon sequestration at some point. ACSR displays the speed of forest carbon accumulation and demonstrate the ability of forest carbon sequestration in a certain period. Therefore, ACSR can express the instantaneous status of forest growth. The ACSR supplies an indication of the proper method to solve this problem.

Developing longtime successive forest inventory data under a warming climate in the future is impossible, and this makes it difficult for landscape models to predict forests dynamics precisely [65], [66]. Applying forest inventory data collected in the field over a short time period with a limited number of data points used to verify the results of our 200-year simulation cannot allow for the creation of an entirely precise model. Comparing simulation results with other studies, including results from other models and experiments, may provide a feasible way to verify the precision of the LANDIS model; this method is sure to enhance the model’s confidence [67].

The analysis of the uncertainty and sensitivity of LANDIS model has been carried out in some studies [26], [43], [68]. The results show that the output of this model is quite stable. The simulated biomass result in this study was also unanimous, and the mean proportional error of the biomass was less than 0.01 (**Fig. 2** **–** **4**). This indicates that the uncertainty of LANDIS model is very low.

One study [69] indicates that the range of biomass accumulation of mixed Korean pine hardwood in the Lesser Khingan Mountains is 199–371 t ha^–1^. Yan *et al*. [70] used the NEWCOP model to simulate the biomass of natural forests in the Lesser Khingan Mountains and found that the value is about 250 t ha^–1^. Our simulation provided a total biomass output of about 250 t ha^–1^ which is in agreement with those previous studies. Moreover, *Fenglin* natural reserve is the largest and oldest reserve in the Lesser Khingan Mountains area. Forests in this reserve have barely obtained damage, and most of the forest communities are in climax state. Therefore, forests in this reserve are considered to be the future state of the forests which are distributed outside the reserve, and the biomass accumulation in reserve is also regarded as the upper limit of forest in the Lesser Khingan Mountains area. The general state of forest in the Lesser Khingan Mountains area 200 years later simulated by LANDIS Pro7.0 model also has been regarded as the climax. We have conducted T-tests among measured biomass data for 157 plots in *Fenglin* natural reserve and calculated each time step’s output of total biomass from 2100 to 2200 in our simulation; the result revealed no significant differences (P>0.05). These facts show that the results of biomass output of the LANDIS model conform to field reality at the landscape scale, and the accuracy of the LANDIS model’s biomass output can be trusted. The LANDIS model was used to evaluate species distribution under possible warming climates in northeast China [17]. The results showed that Korean pine thrives better than other species under warming climatic conditions, and the northern boundary of its range would shift northward while larch decreased under warming climates. The hardwoods were the dominant species in warmer conditions while conifers favored colder circumstances. Our results are consistent with other studies although coniferous species increased in abundance late in our simulation. This simulated effect may occur because the restoration and growth process was slowed by warming climates. Increasing temperature and precipitation would boost the growth rate of sugar maple and white spruce as has been reported in the literature, while the growth rate of balsam fir decreased under the same conditions [71]. Sabedi & Sharma [72] pointed out that the trend of diameter growth in jack pine and black spruce increased and decreased under warming conditions, respectively. Though these two species do not exist in our study area, they provide useful information in evaluating the ACSR of conifers.

In addition, effective comparisons of our results with previous studies such as those discussed above prove the LANDIS model has the ability to simulate forest growth accurately; also, the various results in comparisons provide us with clues for future analysis and modification of the model. The prediction of trends and the alteration of the range of forest ACSR under various climate scenarios in this study tend to agree with previous results.

Great uncertainty exists in climate change predictions of GCMs. Our simulation adopted the climate predictions of CGCM3 because of its applicability in northeastern China that has been proven by many studies [73], [74]. We used only annual mean temperature and precipitation every 10-years and neglected the climate alteration in adjacent years and for a given year; however, this variability could affect the species’ growth, morality, and establishment [75], [76]. Nevertheless, every 10-year average annual temperature and precipitation could reflect the trend of climate change in this area, and the effect of climate change on the forest carbon sequestration rate can also be interpreted appropriately by 10-year increment meteorological data.

In the LANDIS model, species establishment coefficients (SECs) play an important role in deciding the process of forest succession. Future SECs are simulated by the logistics model during different climate change scenarios, and the LANDIS model read the SEC parameters in every time-step to reflect the successive effects of climate change. We care more about forest landscape processes, and some processes do not get enough attention on a more detailed level. However, these processes, such as physiological processes, that are influenced by climate conditions are overlooked while we only use SECs to reflect climate changes indirectly; these physiological processes may influence the biomass accumulation of species. Moreover, biomass equations were used to calculate species’ biomass; however, instability existed in these equations while applying them in different regions.

In this study, species growth curves, as input parameters in LANDIS Pro7.0 model, were obtained from field investigation and previous published references. These results were all based on the historical climate, and the effect of climate condition then had been coupled in the results. Many previous studies [77]–[79] had explored the relationship between tree radial growth and climate conditions; however, we did not conduct similar research, and it might affect the final results of modelling at some extent. In future research, making input parameters more accurate can enhance the credibility of the simulated results.

Furthermore, an important vegetation driver, the fire, was not considered in this study. The regime of fire disturbance would be altered under climate change [80], [81], and it would finally influence forest carbon sequestration. Nonetheless, the purpose of this study was to analysis forest ACSR under different climate change scenarios, and insight was obtained from this research on how forests response to possible climate conditions.

Model simulation is a vital tool in the research of forest dynamics under climate change at long-time and large-scale conditions. Although the model itself has many shortcomings, by appropriately analyzing and verifying the model, user may greatly improve the accuracy of the model. In addition, indigenization of model parameters will produce excellent prospects for successful and accurate application of the model.

## Conclusions

The LANDIS Pro7.0 model is capable of modeling forest dynamics, especially biomass change, under changing climatic conditions. Based on the simulated results, several conclusions can be drawn as follows. Climate warming can influence the ACSR in the Lesser Khingan Mountains area. Mixed Korean pine hardwood forest is the climax community in the Lesser Khingan Mountains area, and it has higher adaption to climate warming when compared with other communities. However, spruce-fir forest has a decline trend while climate becomes warmer. Differences of ACSR of various communities almost emerge around 2140, and this expresses that a hysteresis phenomenon exists in the process of climate change affecting temperate forests ACSR. The ACSR of coniferous species is more strongly influenced by climate change than is the ACSR of deciduous species. The biomass composition of climax communities is not stable, and ACSR can also reflect the response of forest to climate change. The differences of ACSR among various climate change scenarios are complex, and generally, climate change causes the largest impact in the scenario A2.

## Supporting Information

Table S1 **GPS coordinates, vegetation types, and dominant tree species of censored plots in Fenglin nature reserve.**
(XLSX)
